# Pyrolysis Regulation of Agarose into Hierarchical Porous Carbon for Supercapacitor Applications

**DOI:** 10.3390/ma19112298

**Published:** 2026-05-29

**Authors:** Yang Zhao, Mengying Cheng, Siyu Liu, You Wang, Zikun Feng, Wanshi Gu, Yunfeng Guan, Jin Liu, Liya Ma

**Affiliations:** 1 School of Chemistry and Material Science, Hubei Engineering University, Xiaogan 432000, China; 2 Jingzhou Conservation Institute, Jingzhou 434020, China; 3 Core Facility of Wuhan University, Wuhan University, Wuhan 430072, China

**Keywords:** agarose, pyrolysis, supercapacitor

## Abstract

Fundamental understanding of the biomass pyrolysis process on a molecular level provides important guidelines for designing advanced porous carbon materials. In this study, the effects of KOH and K_2_CO_3_ activators on the thermal decomposition of agarose were elucidated using TG-FTIR-GCMS coupling techniques. The results demonstrate that the presence of KOH/K_2_CO_3_ shifts the pyrolysis gaseous products from organic fragments to CO_2_ and H_2_O, thereby preserving more C-C bonds in the solid phase and facilitating the subsequent aromatization process. Furthermore, compared to using KOH as the sole activator, the K_2_CO_3_/KOH co-activation strategy suppresses the violent evolution of CO_2_ within the 300–400 °C range, thereby alleviating the structural shock to the material skeleton and ensuring its integrity. Therefore, the HPC-KCO prepared via a synergistic KOH/K_2_CO_3_ co-activation and one-step carbonization process exhibits a high specific surface area of 1670 m^2^ g^−1^ and successfully retains its interconnected hierarchical porous framework. Benefiting from its well-developed porous structure, HPC-KCO exhibits an impressive specific capacitance of 370 F g^−1^ when employed in zinc-ion capacitors. Furthermore, the assembled symmetric supercapacitor demonstrates robust stability over a wide temperature range from −60 to 100 °C, delivering a remarkable capacitance of 121 F g^−1^ even at −60 °C. This work offers a new insight for synthesizing porous structures of biomass-derived carbon.

## 1. Introduction

Supercapacitors serve as a technological bridge connecting the characteristics of conventional dielectric capacitors and rechargeable batteries [1]. Owing to a series of excellent characteristics, including high power output, fast charge/discharge rates, long cycle life, broad operating temperature ranges, and intrinsic safety, supercapacitors have attracted widespread research attention in energy storage research [2]. For practical applications, porous carbons are overwhelmingly utilized to fabricate the electrodes. This preference is driven by the materials’ exceptionally high surface area, well-developed hierarchical pores, excellent conductive networks, and strong chemical stability [3]. Therefore, innovating sophisticated porous carbon materials is unequivocally the critical bottleneck to further elevating supercapacitor performance.

As a highly abundant and renewable resource, biomass encompasses a wide range of organic matter sourced from plants, animals, and marine environments [4,5]. Researchers have long utilized these materials across diverse sectors, including fuel production, chemical extraction, and the development of functional materials [6]. Beyond these conventional applications, the naturally high carbon content of biomass positions it as an exceptional precursor for fabricating carbon-based electrodes in supercapacitor [7].

Agarose, a linear polysaccharide biomass extracted from red algae, consists of alternating 1,3-linked β-D-galactose and 1,4-linked 3,6-anhydro-α-L-galactose units. This precise configuration allows the polymer to dissolve in hot water and undergo stable gelation upon cooling [8,9]. Zhang et al. synthesized activated carbon by a simple one-step calcination of deoxygenated agar in a hot KOH aqueous solution, in which KOH plays both deoxidant and activation agent. The deoxygenation course leads to molecular level activation of agar in calcination [10]. The obtained porous carbon electrode exhibited a specific surface area of up to 1672 m^2^ g^−1^. However, it delivered a moderate specific capacitance of only 220 F g^−1^. This is mainly because the synthesized carbon features a highly microporous structure, resulting in inadequate diffusion channels for electrolyte ions.

In previous studies, researchers have explored various preparation methods, including chemical activation, hydrothermal carbonization and templating to fabricate biomass-derived carbon with hierarchical porous structure [11,12,13,14]. For example, Yi et al. synthesized an N, O, and S co-doped hierarchical porous carbon using sodium lignosulfonate and sodium humate as precursors [15]. By coupling boric acid templating with KOH activation, the material achieved a specific surface area of 1588 m^2^ g^−1^ and demonstrated a specific capacitance of 318 F g^−1^ in KOH electrolyte. Furthermore, Li et al. utilized chitosan as a carbon source and combined an ice-templating strategy with KOH activation to prepare carbon foams featuring a hierarchical porous network [16]. The multiscale pore network facilitates the fast-charging capability of the carbon electrodes. However, a common limitation of these methods is their reliance on a two-step process with carbonation and activation. This is primarily because the one-step process often makes hierarchical pore architecture highly susceptible to structural collapse [17].

Deciphering biomass pyrolysis at the molecular level facilitates the precise design of hierarchical porous carbon materials. Although previous studies have utilized in-situ DRIFTS to track gas evolution [18], the cumulative nature of reaction products makes it difficult to link these species with specific heating stages and to accurately identify the exact types of decomposition products. The integrated TG-FTIR-GCMS system holds promise for addressing this issue by enabling the real-time synchronization of mass-loss data with both the vibrational and qualitative mass spectra of evolved gases. Thus, this capability provides an approach for investigating the role of activators during the biomass pyrolysis and carbonization process.

This study presents an integrated approach combining a freeze-drying templating method with a synergistic K_2_CO_3_/KOH co-activation strategy to synthesize hierarchical porous carbons from an agarose precursor via a one-step high-temperature treatment. By employing the integrated TG-FTIR-GCMS technique, we analyzed the decomposition species evolved during the programmed heating process to elucidate the specific regulatory roles of the activators. The analytical results indicate that this co-activation strategy preferentially preserves carbon–carbon bonds within the 200–300 °C range, thereby facilitating subsequent aromatization reactions. Concurrently, K_2_CO_3_ acts as a kinetic buffer that moderates the violent release of carbon dioxide typically observed during pure KOH activation, thus preventing the collapse of the carbon framework. As a result, the as-prepared HPC-KCO material exhibits a high specific surface area of 1670 m^2^ g^−1^ while successfully retaining the multi-level hierarchical pore structure generated by the templating process. When assembled into a zinc-ion capacitor, the HPC-KCO electrode delivers an ultrahigh specific capacitance of 370 F g^−1^. Furthermore, symmetric supercapacitors (SSCs) based on HPC-KCO demonstrate stable energy storage capabilities across an ultra-wide temperature range, achieving a specific capacitance of 121 F g^−1^ at −60 °C.

## 2. Experiment Section

### 2.1. Materials

Potassium hydroxide (KOH, AR), potassium carbonate (K_2_CO_3_, AR), hydrochloric acid (HCl, AR), and absolute ethanol (AR), were purchased from Sinopharm Chemical Reagent Co., Ltd. (Shanghai, China). Agarose, tetraethylammonium tetrafluoroborate (TEA-BF_4_), 3-methoxypropionitrile (MPN), polytetrafluoroethylene (PTFE, 60 wt%) and zinc sulfate (ZnSO_4_) were purchased from Aladdin Reagent Co., Ltd. (Shanghai, China). All aqueous solutions were prepared using deionized water, and all chemicals were employed without further purification.

### 2.2. Synthesis of HPCs

In a typical synthesis, 1.0 g agarose, 0.2 g K_2_CO_3_, and 0.6 g KOH were dissolved in 20 mL deionized water under vigorous stirring at 100 °C for 20 min to form a homogeneous solution. The solution was cooled to room temperature to form a hydrogel, followed by freeze-drying. The freeze-dried sample was then carbonized in a tubular furnace under Ar flow, heated to 900 °C at a ramp rate of 5 °C min^−1^, and maintained for 2 h before cooling to room temperature. The carbonized product was washed three times with deionized water and dried overnight at 80 °C to obtain the porous carbon material, denoted as HPC-KCO. For comparison, HPC-KO (with 0.8 g KOH only) and HPC-KC (with 0.8 g K_2_CO_3_ only) were prepared under identical conditions.

### 2.3. Material Characterization

The morphology and microstructure of the materials were characterized by field-emission scanning electron microscopy (FESEM, Sigma, Zeiss, Oberkochen, Germany) and field-emission transmission electron microscopy (FETEM, JEM-F 200, JEOL, Tokyo, Japan). Surface composition analysis was performed using X-ray photoelectron spectroscopy (XPS, ESCALAB 250 Xi, Thermo Fisher, Waltham, MA, USA). Nitrogen adsorption–desorption isotherms were measured at 77 K with a TriStar II 3020 analyzer (Micromeritics, Norcross, GA, USA), Crystalline structures were analyzed by X-ray diffraction (XRD, X’pert PRO, Panalytical, Almelo, The Netherlands) with Cu Kα radiation (λ = 1.5406 Å). Raman spectroscopy (XploRA Plus, HORIBA JobinYvon, Longjumeau, France) was conducted using a 532 nm laser within the 50–2500 cm^−1^ range for phase identification. The TG/FTIR/GCMS were carried out by TGA8000/Spectrum3/Clarus 690/Clarus SQ8T (Perkin Elmer, Waltham, MA, USA), the sample was heated from 30 to 800 °C at a heating rate of 5 °C per minute under Ar atmosphere. The thermal stability of the MPN electrolyte was measured by a differential scanning calorimeter (DSC, Mettler, Columbus, OH, USA) over a temperature range of −40 °C to 100 °C.

### 2.4. Electrochemical Measurements

The working electrodes were prepared by thoroughly mixing the as-synthesized carbon material, polytetrafluoroethylene (PTFE) binder, and conductive carbon black in an 8:1:1 mass ratio. The mixture was ground in an agate mortar until a homogeneous slurry. The slurry was then uniformly coated onto a stainless steel mesh current collector. The three-electrode measurements were conducted in a 6 M KOH electrolyte with an HgO/Hg reference electrode and a Pt counter electrode. For ZIHCs, Zn foil and 2 M ZnSO_4_ were used as the anode electrode and electrolyte, respectively. Consistent with prior studies, the ZISC was operated within a voltage range of 0.1 to 1.8 V [19]. For the wide-temperature SSC device, two prepared electrodes were assembled into a symmetric supercapacitor using a glass fiber separator. The electrolyte consisted of TEA-BF_4_ dissolved in MPN and was employed as a wide-temperature electrolyte. The device was then tested across a temperature range of −60 °C to 100 °C. Drawing from our prior research, the voltage window for the SSC was set at 2.5 V for −60 °C and 1.5 V for 100 °C, respectively [20]. The tests at low temperatures were operated in a DW-138 low temperature test chamber (Zhongke Meiling Cryogenics Co., Ltd., Hefei, China).

For the supercapacitor, the specific capacitance was formulated as followed:

(1) $$C_{S,CV}=\frac{\int IdV}{mv\Delta V}$$

(2) $$C_{S,GCD}=2\frac{I\Delta t_{d}}{m\Delta V}$$

The m (g) is the mass of active material on a single electrode.

The energy density (E_wt_, Wh·kg^−1^) and power density (P*_wt_*, W·kg^−1^) of SSCs were accordingly calculated by equations:

(3) $$E_{wt}=\frac{1}{2\times 4\times 3.6}C_{s}\Delta V^{2}$$

(4) $$P_{wt}=\frac{3600\times E_{wt}}{\Delta t}$$

## 3. Results and Discussion

**Figure 1:**
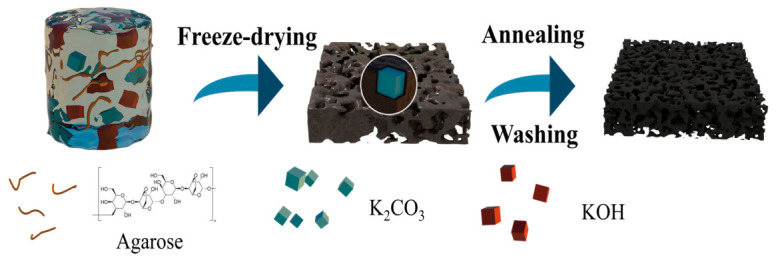
Schematic illustration for HPC-KCO.

The synthesis procedure for HPC-KCO is schematically illustrated in Figure 1. The process begins with the dissolution of the agarose precursor, along with K_2_CO_3_ and KOH, in deionized water to form a homogeneous hydrogel. Subsequently, the hydrogel is subjected to freeze-drying, wherein the sublimating ice crystals act as a template to construct an initial porous architecture. Finally, the resulting aerogel undergoes high-temperature carbonization under an inert atmosphere, followed by washing and drying, to yield the target hierarchical porous carbon material.

**Figure 2:**
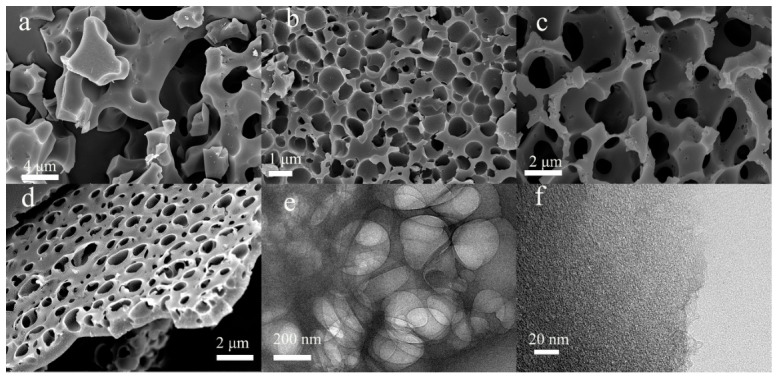
The SEM images of HPC-KO (**a**), HPC-KC (**b**) and HPC-KCO (**c**,**d**). TEM image (**e**) and HRTEM image (**f**) of HPC-KCO. The scale bar is 4 μm (**a**), 1 μm (**b**), 2 μm (**c**,**d**), 200 nm (**e**) and 20 nm (**f**), respectively.

The microstructural evolution of carbon materials was systematically investigated through combined microscopic analyses. As evidenced by SEM observations (Figure 2), the pore architecture exhibited strong dependence on activator composition. The K_2_CO_3_-free sample (HPC-KO) exhibited a nonporous monolithic morphology without detectable pore structures (Figure 2a). Upon the incorporation of K_2_CO_3_, both HPC-KC and HPC-KCO developed three-dimensional porous frameworks due to the templating effect of K_2_CO_3_. Furthermore, HPC-KCO demonstrated enhanced pore interconnectivity resulting from the synergistic etching between KOH and K_2_CO_3_ (Figure 2b,c).

The freeze-drying process effectively utilized ice crystals as structural templates to construct lamellar architectures (Figure 2d and Figure S1), which facilitated electrolyte permeation [21]. TEM was also used to investigate the structures of the synthesized materials. HPC-KO exhibits a dense block-like structure (Figure S2), whereas the structure of HPC-KCO contains abundant submicron pores (Figure 2e). Meanwhile, high-resolution transmission electron microscopy (HRTEM) images reveal abundant micropores within its carbon framework (Figure 2f).

**Figure 3:**
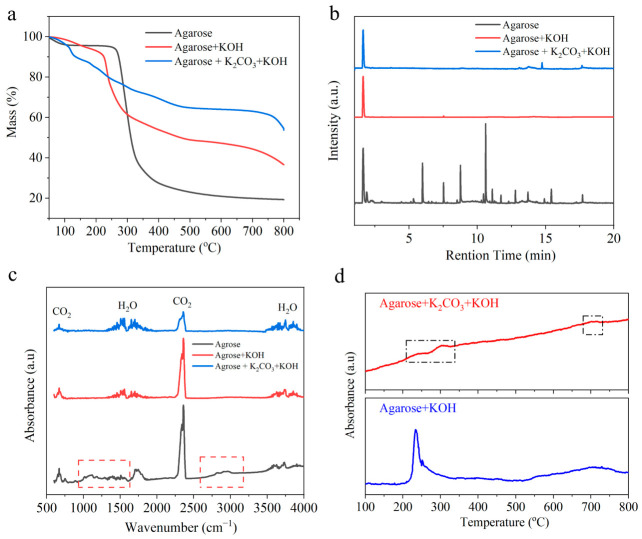
TG curves of agarose during temperature-programmed heating from 30 to 800 °C (**a**), gas chromatograms at 280 °C (**b**) and infrared spectrums of the decomposition products (**c**). The dependences of infrared absorption intensity of evolved CO_2_ on temperatures (**d**).

To further clarify the effects of K_2_CO_3_ and KOH on the pyrolysis synthesis of agarose-derived carbon, we employed TG/FTIR/GCMS to investigate the pyrolysis behavior and product characteristics of agarose gel. As shown in Figure 3a, Pure agarose remains stable up to 260 °C, followed by a sharp mass loss (260–400 °C) due to the intensive pyrolysis of polymer chains and glycosidic bond cleavage. Beyond 400 °C, a slow mass reduction corresponds to carbonization and aromatization. In contrast, the addition of KOH significantly lowers the decomposition onset to ~220 °C, indicating its catalytic role in promoting the premature cleavage of glycosidic bonds and accelerated dehydration. Notably, the KOH/K_2_CO_3_ dual-activator system modifies this pathway further: the vertical mass drop observed in pure agarose is replaced by a gradual 45° slope between 200 and 450 °C. This indicates that the dual activator system can make the pyrolysis process of agarose more moderate, favoring the formation of a more stable carbon skeleton [18,22].

The gaseous products evolved at 280 °C were further analyzed by GCMS and FTIR. As shown in Figure 3b, pure agarose decomposes into complex organic fragments, including levoglucosan, furan derivatives and hydrocarbon products (Table S1). This indicates that the remarkable weight loss of agarose at 300–400 °C is caused by the random cleavage of glycosidic bonds and the overall fragmentation of the polysaccharide backbone. In this stage, the polymer chains undergo disordered decomposition to form large monomeric or dimeric fragments, which escape rapidly from the solid phase, resulting in a drastic mass loss. The FTIR spectra (Figure 3c) show characteristic absorption peaks corresponding to organic functional groups such as C–O, C=O, and C–H, which are consistent with the GC-MS results. In contrast, for the samples modified with KOH and KOH/K_2_CO_3_, the organic signals in both GCMS and FTIR spectra disappear completely, and only the characteristic absorptions of CO_2_ and H_2_O are observed in the gaseous products. Meanwhile, the C–C bonds in agarose are largely preserved, enabling the aromatization transformation at higher temperatures.

The evolution kinetics of CO_2_ were further tracked from 100 to 800 °C (Figure 3d) to elucidate the activator-dependent morphological evolution. Pure KOH induces an aggressive, stage-wise reaction with a sharp CO_2_ burst at 200–300 °C and a secondary activation peak at 700–800 °C. This explosive gas release, coupled with intensive chemical etching, creates high internal pressure that collapses the fragile, lyophilized lamellar structure of the agarose precursor. In stark contrast, the KOH/K_2_CO_3_ system exhibits a steady, monotonic increase in CO_2_ emission, closely resembling the continuous trend of pure agarose but without organic volatiles (Figure 3d and Figure S3). Here, K_2_CO_3_ acts as a kinetic regulator that buffers the reactivity of KOH, transforming the violent decomposition into a tempered, sustained activation process. This controllable kinetic pathway enables the carbon skeleton to solidify along the K_2_CO_3_ crystal structure at 300–400 °C without structural collapse, while successfully preserving the porous lamellar morphology inherited from the freeze-dried gel.

**Figure 4:**
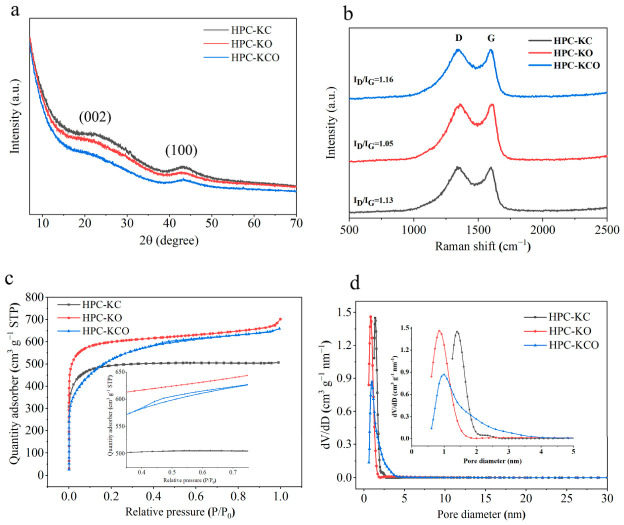
XRD patterns (**a**), Raman spectrums (**b**), nitrogen adsorption–desorption isotherms (**c**) and pore size distributions (**d**) of obtained HPC samples.

The X-ray diffraction patterns (Figure 4a) exhibited broad diffraction peaks centered at 2θ ≈24° (002 plane) and 43° (100 plane), characteristic of graphitic stacking structures and in-plane crystallographic ordering, respectively [23]. The significant peak broadening suggests the amorphous nature of the carbon matrices. Raman spectral analysis (Figure 4b) revealed two distinct vibrational modes: a D-band at 1350 cm^−1^ (associated with disordered carbon domains) and a G-band at 1580 cm^−1^ (attributed to graphitic sp^2^-bonded carbon) [24]. The calculated _ID_/I_G_ ratios of 1.13 (HPC-KC), 1.05 (HPC-KO), and 1.16 (HPC-KCO) reveal two key structural characteristics. The samples exhibit comparable graphitization levels, which is consistent with their shared agarose precursor and identical 900 °C carbonization temperature [25].

Nitrogen physisorption analysis revealed distinct hierarchical porosity across the synthesized carbons. As shown in Figure 4c, all samples exhibit a strong adsorption capacity in the relative pressure range of 0 < P/P_0_ < 0.01, demonstrating that the three samples possess abundant micropores after activation [2]. Additionally, HPC-KCO exhibits a combined Type I/IV nitrogen adsorption–desorption isotherm, with a hysteresis loop observed in the P/P_0_ range of 0.4–0.8, confirming the presence of mesopores in its structure (the inset of Figure 4c). In contrast, no hysteresis loops are observed for HPC-KO and HPC-KC, indicating that their pore structures are predominantly microporous [12]. Pore size distributions (Figure 4d) displayed steep peaks at 1.0–1.5 nm for all specimens, while HPC-KCO uniquely demonstrated asymmetric tailing extending to 3 nm, indicative of mesopore formation through partial micropore coalescence during the KOH/K_2_CO_3_ dual-activation process. This synergistic mechanism involved KOH-induced micropore generation via oxidative etching combined with CO_2_ release from K_2_CO_3_ decomposition, which synergistically promoted pore-widening effects and structural reorganization. As shown in Table S2, HPC-KO achieved maximal specific surface area (1859.6 m^2^ g^−1^) with 0.58 cm^3^ g^−1^ micropore volume, whereas HPC-KCO showed reduced micropore volume (0.40 cm^3^ g^−1^) and specific surface area (SSA, 1670 m^2^g^−1^). As shown in Table S2, HPC-KO achieved a maximum specific surface area (SSA) of 1859.6 m^2^g^−1^ with a micropore volume of 0.58 cm^3^ g^−1^. In contrast, HPC-KCO exhibited a reduced micropore volume (0.40 cm^3^ g^−1^) and SSA (1670 m^2^ g^−1^). However, the total pore volumes of both samples remained similar, suggesting that the co-activation with K_2_CO_3_ and KOH favors the formation of larger pores. This is further supported by the pore size distribution (PSD) in Figure 4d, where HPC-KO pores are predominantly centered around 2 nm, whereas HPC-KCO shows a broader distribution within the 2–4 nm range. On the other hand, compared to HPC-KC (SSA: 1542 m^2^ g^−1^; pore volume: 0.78 cm^3^ g^−1^), HPC-KCO exhibits enhanced textural properties, indicating that the synergistic effect of K_2_CO_3_ and KOH activation is more conducive to developing a well-developed porous structure. The microstructural analysis, combining SEM and TEM observations, confirms that HPC-KCO possesses an interconnected pore structure. This hierarchical porous architecture effectively bridges the primary micropores, thereby optimizing mass transport while maintaining excellent surface accessibility [2].

XPS analysis confirmed the surface chemical composition of the carbon materials, exclusively revealing carbon and oxygen species (Figure S4a). As shown in Figure S4b, the C 1s spectrum deconvoluted into three components: a dominant peak at 284.8 eV (C-C/C=C sp^2^ hybridization), with minor contributions at 286.6 eV (C-O bonds) and 289.8 eV (carboxyl groups, C=O) [19]. Correspondingly, the O 1s spectrum (Figure S4c) exhibited two characteristic peaks at 531.6 eV (quinone-type C=O) and 532.8 eV (epoxy/hydroxyl C-O), confirming the coexistence of both acidic and basic oxygen functionalities [26]. These oxygen-containing groups, synergistically combined with the hierarchical porosity and defect-rich graphitic domains characterized earlier, likely enhance interfacial wettability [27].

First, the electrochemical performance of the synthesized materials was evaluated using a three-electrode system. The test results are shown in Figure S5, where HPC-KCO exhibits good capacitive performance, reaching a specific capacitance of 356 F g^−1^ at a current density of 1 A g^−1^. Even at an ultrahigh current density of 50 A g^−1^, it still maintains a specific capacitance of 224 F g^−1^. Meanwhile, it also demonstrates favorable electrochemical stability during cycling.

**Figure 5:**
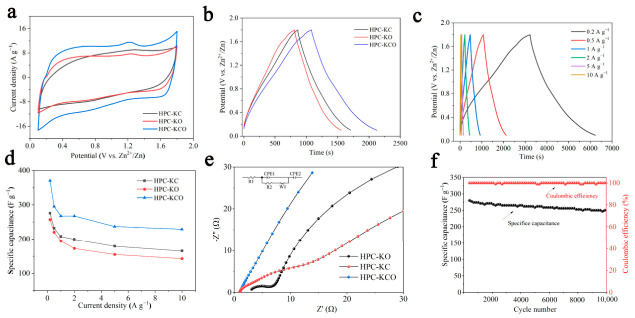
CV curves (**a**) and GCD curves (**b**) of Zn//HPC. GCD curves at 0.5–10 A g^−1^ of Zn//HPC-KCO (**c**); Corresponding specific capacitance of the Zn//HPC devices as a function of current densities (**d**); electrochemical impedance spectroscopy (**e**); Cycling stability of the assembled device over 10,000 cycles at a current density of 5 A g^−1^ (**f**).

To demonstrate the practical potential of the synthesized materials, a zinc-ion hybrid supercapacitor (ZISC) was fabricated. The operating voltage window was optimized between 0.1 and 1.8 V to ensure electrolyte stability and suppress parasitic side reactions [19]. The capacitive performance of the assembled ZISC was investigated through cyclic voltammetry (CV) tests at a scan rate of 50 mV s^−1^ (Figure 5a) and galvanostatic charge–discharge (GCD) tests at a current density of 0.5 A g^−1^ (Figure 5b). As shown in Figure 5a, the CV curve of the Zn//HPC-KCO exhibits a larger enclosed area compared to those of the HPC-KO and HPC-KC based hybrid capacitors, indicating a superior charge storage capacity [28]. Furthermore, the GCD test results in Figure 5b demonstrate that the Zn//HPC-KCO capacitor displays the longest charge–discharge curve among the three assembled hybrid capacitors, indicating its superior specific capacitance. Additionally, all CV curves exhibit quasi-rectangular shapes, while the GCD curves show quasi-isosceles triangular profiles, deviating slightly from the standard symmetric linear shape characteristic of electric double-layer capacitors [29,30].

Figure 5c and Figure S6 display the GCD profiles of all samples at various current densities. The highly symmetrical triangular shapes of the GCD curves, which are well-maintained even as current density increases, demonstrate excellent electrochemical reversibility and ideal capacitive behavior. Figure 5d compares the specific capacitance retention of the different hybrid supercapacitors. At 0.2 A g^−1^, HPC-KC, HPC-KO, and HPC-KCO achieve maximum specific capacitances of 275.6, 257, and 370 F g^−1^, respectively. When the current density is increased to 10 A g^−1^, HPC-KCO maintains a specific capacitance of 233 F g^−1^ with a 62% retention rate, outperforming HPC-KC (165 F g^−1^, 59%) and HPC-KO (143 F g^−1^, 56%). This enhanced performance suggests that HPC-KCO, synthesized via the synergistic activation of K_2_CO_3_ and KOH, possesses superior rate capability for zinc-ion hybrid supercapacitors.

Electrochemical impedance spectroscopy (EIS) was employed to further investigate the electrochemical performance and charge-transfer kinetics of the prepared electrodes (Figure 5e). The charge transfer resistance (Rct) of the electrodes is represented by the diameter of the semicircle in the high-to-medium frequency region of the Nyquist plot, while the equivalent series resistance (Rs) is determined by the intercept of the plot on the real axis at the high-frequency limit [28]. For zinc-ion capacitors, HPC-KCO exhibits the lowest Rs (0.5 Ω) and Rct (1.2 Ω), which are notably lower than those of HPC-KO (Rs of 2.6 Ω and Rct of 2.8 Ω) and HPC-KC (Rs of 0.9 Ω and Rct of 2.0 Ω). This demonstrates that the HPC-KCO electrode facilitates rapid zinc-ion diffusion and significantly enhances the overall capacitive performance. Furthermore, the slope of the HPC-KCO curve in the low-frequency region is closer to 90°, which indicates minimal diffusion resistance and efficient electrolyte ion transport into the electrode [31]. As shown in Figure 5f, the Zn//HPC-KCO device demonstrates exceptional long-term cycling stability at a high current density of 5 A g^−1^. After 10,000 cycles, the specific capacitance decreases slightly from 282 F g^−1^ to 270 F g^−1^, maintaining a capacity retention of 88%. Furthermore, the Coulombic efficiency remains near 100% throughout the entire cycling process, signifying exceptional electrochemical reversibility and structural robustness of the electrode. This outstanding long-term durability further validates the stability and appropriateness of the selected operating voltage window for the as-assembled device.

**Figure 6:**
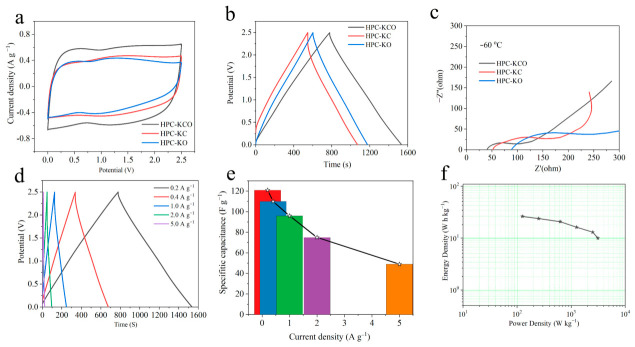
CV curves (**a**), GCD curves (**b**) and Nyquist plots (**c**) of SSCs with different electrodes at −60 °C; GCD curves (**d**) and corresponding specific capacitance (**d**) of SSC based on HPC-KCO under various current density at −60 °C. (**e**) Corresponding specific capacitance of the HPC-KCO- at −60 °C. (**f**) The Ragone plots of the SSC based on HPC-KCO.

In practical applications, supercapacitors are frequently subjected to harsh environmental temperatures. Therefore, to evaluate the capacitive performance under extreme conditions, we assembled symmetric supercapacitors using the HPC electrodes and a wide-temperature-range electrolyte, and tested them across a broad temperature range from −60 to 100 °C. The voltage window of this electrolyte has been verified in our previous work [20]. The test result demonstrates that this system can withstand potentials up to 3.0 V at low temperatures. Thus, a stable operating window of 2.5 V was adopted for low-temperature testing. As depicted in Figure 6a, the CV curves of the three samples, measured at −60 °C with a scan rate of 10 mV s^−1^, reveal that the HPC-KCO electrode exhibits the largest integrated area, indicative of its superior specific capacitance. Corroborating these CV findings, the GCD tests at 0.2 A g^−1^ (Figure 6b) show a consistent trend. The HPC-KCO electrode delivers the longest discharge time, signifying its outstanding capacitive performance. Furthermore, the highly symmetrical triangular profile observed in the GCD curves of the HPC-KCO electrode suggests characteristic electric double-layer capacitive (EDLC) behavior [32]. Figure 6c displays Nyquist plots wherein HPC-KCO exhibits the smallest Rs and Rct, derived from the semicircle diameter among the samples. The slope in the low-frequency region deviates slightly from 90°; this phenomenon is primarily attributed to the hindered ion migration rates and sluggish kinetics caused by the low-temperature environment. Figure 6d illustrates the GCD curves of HPC-KCO at −60 °C under varying current densities (0.2–5 A g^−1^), with the corresponding specific capacitances calculated from these profiles being systematically compared in Figure 6e. Under cryogenic conditions of −60 °C, the HPC-KCO electrode demonstrates a remarkable specific capacitance of 121 F g^−1^ at 0.2 A g^−1^. Even at 5 A g^−1^, a capacitance of 46 F g^−1^ is preserved. These characteristics arise from the unique lamellar architecture that ensures thorough electrolyte infiltration, coupled with a 3D porous framework that constructs continuous ion-conducting pathways to facilitate interfacial ion transfer [33]. Figure 6f presents the Ragone plot of the HPC-KCO based supercapacitor, where energy densities of 26.2 Wh kg^−1^ at 125 W kg^−1^ and 10.0 Wh kg^−1^ at 3125 W kg^−1^ are achieved under −60 °C operation. Remarkably, this performance surpasses most recently reported cryogenic supercapacitors, demonstrating exceptional energy–power balance in extreme environments [31,34,35,36,37,38]. Furthermore, the device maintains 80% capacitance retention after 10,000 cycles at 2 A g^−1^ under −60 °C, demonstrating exceptional cyclic durability in cryogenic environments (Figure S7).

**Figure 7:**
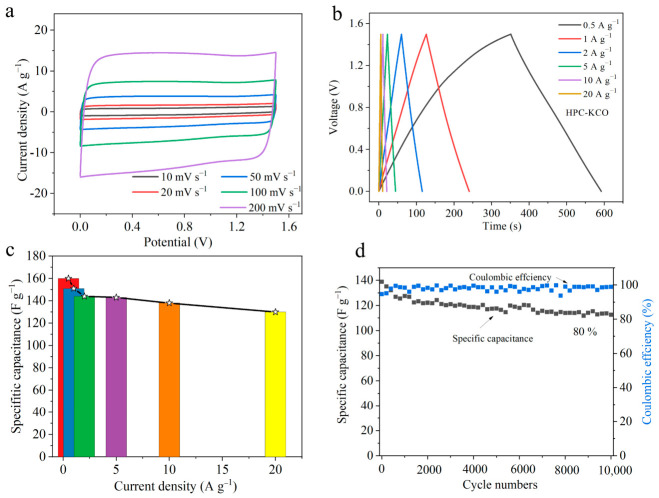
The CV curves (**a**) and GCD curves (**b**) of the SSC assembled with HPC-KCO at 100 °C; Corresponding specific capacitance of the HPC-KCO-based supercapacitor at 100 °C; (**c**,**d**) cycling stability of the SSC using HPC-KCO over 10,000 cycles at 100 °C.

As depicted in Figure S8, the DSC curve reveals that no endothermic peak appears even when the electrolyte is heated up to 100 °C. This observation demonstrates that no phase transition occurs in the electrolyte at this elevated temperature. At elevated temperatures, the operating voltage window of the supercapacitor must be appropriately narrowed compared to ambient conditions to mitigate electrolyte degradation and suppress parasitic reactions [39]. Based on our preliminary findings, CV profiles of MPN-based electrolytes exhibit noticeable distortion at 100 °C when the potential exceeds 1.6 V, signaling the onset of parasitic side reactions. Consequently, the operating voltage window in this work was restricted to 1.5 V to ensure long-term electrochemical stability under high-temperature conditions [20]. As illustrated in Figure 7a, the CV curves of the SSC maintain a quasi-rectangular profile across all scan rates. This signifies ideal capacitive behavior and confirms the stable operation of the device within the designated voltage window [40].

Figure 7b presents the GCD curves of HPC-KCO measured at 100 °C, with corresponding specific capacitances calculated from these profiles being systematically tabulated in Figure 7c. The linear voltage–time relationships observed across all current densities confirm ideal capacitive behavior with minimal IR drop during high-temperature operation. The HPC-KCO electrode delivers a specific capacitance of 150 F g^−1^ at 1 A g^−1^, with 130 F g^−1^ retained at 20 A g^−1^ (86.7% capacity retention), demonstrating exceptional rate capability. The remarkable rate capability arises from its unique macroporous–lamellar architecture, where micrometer-scale pores facilitate rapid electrolyte infiltration while the continuous conductive networks ensuring efficient electron transport throughout charge–discharge processes [41]. Furthermore, the symmetric supercapacitor assembled using HPC-KCO exhibits remarkable durability even at an elevated temperature of 100 °C. At a high current density of 20 A g^−1^, the device retains 80% of its initial specific capacitance after 10,000 cycles. The Coulombic efficiency consistently stays near 100% throughout the process, demonstrating the superior thermal stability and robust electrochemical reversibility of the electrode framework (Figure 7d).

## 4. Conclusions

In summary, we have successfully developed a novel dual-templating strategy, synergistically coupling ice-crystal templating with K_2_CO_3_/KOH co-activation, to fabricate high-performance hierarchical porous carbon (HPC-KCO) from an agarose precursor. TG/FTIR/GCMS characterization revealed that the dual-activator system fundamentally moderates the thermal decomposition pathway of agarose. Unlike the severe fragmentation of the pure precursor, this approach suppresses the random cleavage of glycosidic bonds, instead promoting the preferential cleavage of C-O bonds to form H_2_O. This unique mechanism preserves the C-C backbone, facilitating the construction of a porous carbon skeleton. This meticulously engineered process yields an HPC-KCO with a highly interconnected 3D hierarchical porous network, optimized for both rapid ion transport and high-capacity charge storage. Consequently, these structural advantages translate directly into outstanding electrochemical performance. As a demonstration, the HPC-KCO not only delivers a high specific capacitance of 370 F g^−1^ as a zinc ion hybrid capacitor cathode but also enables a symmetric supercapacitor with remarkable stability across a wide temperature range from −60 to 100 °C. This work pioneers a new idea for rationally designing porous carbons by regulating the pyrolysis process of biomass precursors.

## Data Availability

The original contributions presented in this study are included in the article/Supplementary Materials. Further inquiries can be directed to the corresponding authors.

## Supplementary Materials

The following supporting information can be downloaded at: https://www.mdpi.com/article/10.3390/ma19112298/s1, Figure S1: The SEM image of HPC-KCO; Figure S2: The TEM image of HPC-KO; Figure S3: The dependence of infrared absorption intensity of CO_2_ released from agarose on temperature; Figure S4: XPS spectra of HPC (a); High-resolution XPS C 1s and (b) O 1s (c) spectra of HPC-KCO; Figure S5: (a) CV curves of the HPC electrodes at 100 mV s^−1^. (b) GCD curves at 1 A g^−1^. (c) GCD curves of the HPC-KCO electrode at 1–50 A g^−1^. (d) Specific capacitance of the HPC-KCO electrodes at 1–50 A g^−1^. (e) Cycling stability of the HPC-KCO electrode at 10 A g^−1^. The measurement were tested in a three-electrodes system; Figure S6: The GCD curves of Zn//HPC assembled with HPC-KO (a) and HPC-KC (b); Figure S7: The cycling stability of the SSC using HPC-KCO over 10,000 cycles under −60 °C; Figure S8: The DSC curves of the MPN-based electrolyte in the temperature range of −40 °C to 100 °C; Table S1: GC/MS Library Search Report of Gaseous Products from Agarose Pyrolysis at 280 °C; Table S2: Textural parameters of samples using BET model.
