# Supplementary material for: Pyrolysis Regulation of Agarose into Hierarchical Porous Carbon for Supercapacitor Applications
Source: Materials (Basel). 2026 May 29;19(11):2298. doi: 10.3390/ma19112298 (PMC13258260; doi:10.3390/ma19112298)
Supplement: Supplementary file 1 [file materials-19-02298-s001.zip › materials-4306442-supplementary.pdf]

Pyrolysis Regulation of Agarose into Hierarchical Porous
Carbon for Supercapacitor Applications

Yang Zhao [1,2], Mengying Cheng [3], Siyu Liu [1], You Wang [1], Zikun Feng [1], Wanshi Gu [1], Yunfeng Guan [1], Jin Liu [1,*] and Liya Ma [3,*]

[1] School of Chemistry and Material Science, Hubei Engineering University, Xiaogan 432000, China;
zhaoyangwhu@126.com (Y.Z.); 18771773058@163.com (S.L.);
19572299061@163.com (Y.W.);
fengzikun330@163.com (Z.F.); 13147294288@163.com (W.G.);
guanyunfeng@hbeu.edu.cn (Y.G.)
[2] Jingzhou Conservation Institute, Jingzhou 434020, China
[3] Core Facility of Wuhan University, Wuhan University, Wuhan 430072, China;
c2432131476@126.com
* Correspondence: liu.j@hbeu.edu.cn (J.L.); maliya@whu.edu.cn (L.M.)

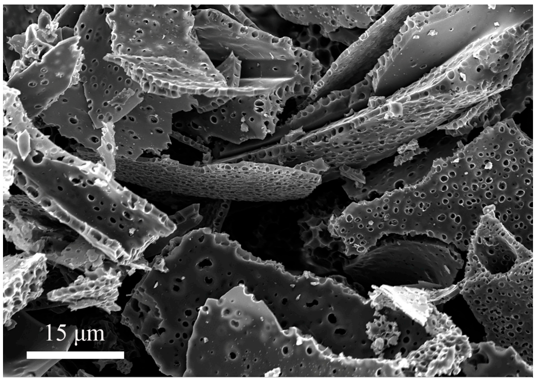

Figure S1 The SEM image of HPC-KCO

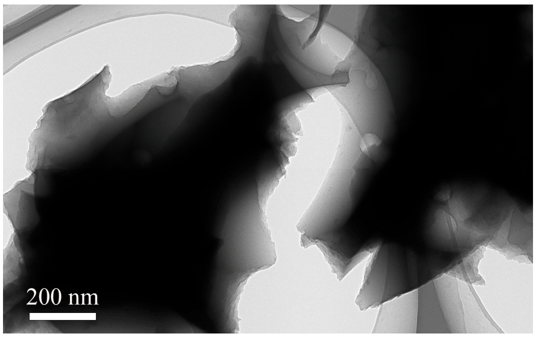

Figure S2 The TEM image of HPC-KO

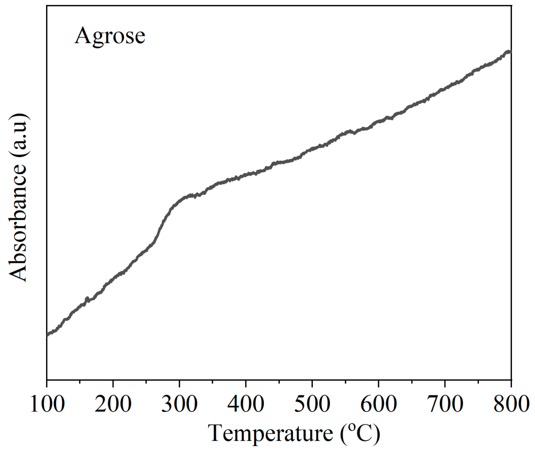

Figure S3 The dependence of infrared absorption intensity of CO_2 released from agarose on temperature

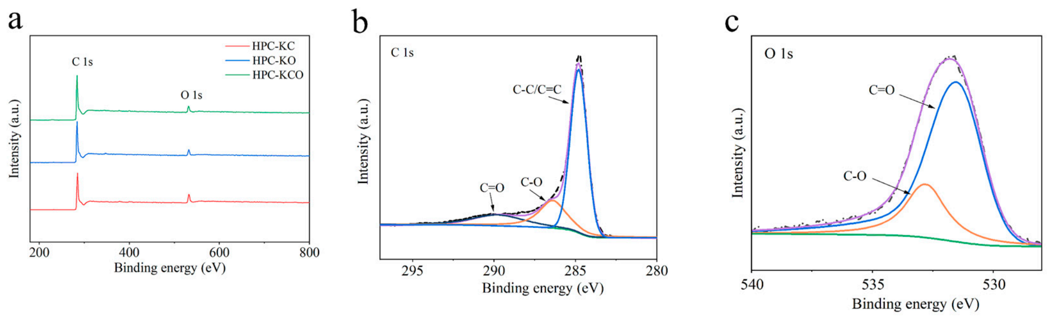

Figure S4 XPS spectra of HPC(a); High-resolution XPS C 1s and (b) O 1s (c)spectra of HPC-KCO.

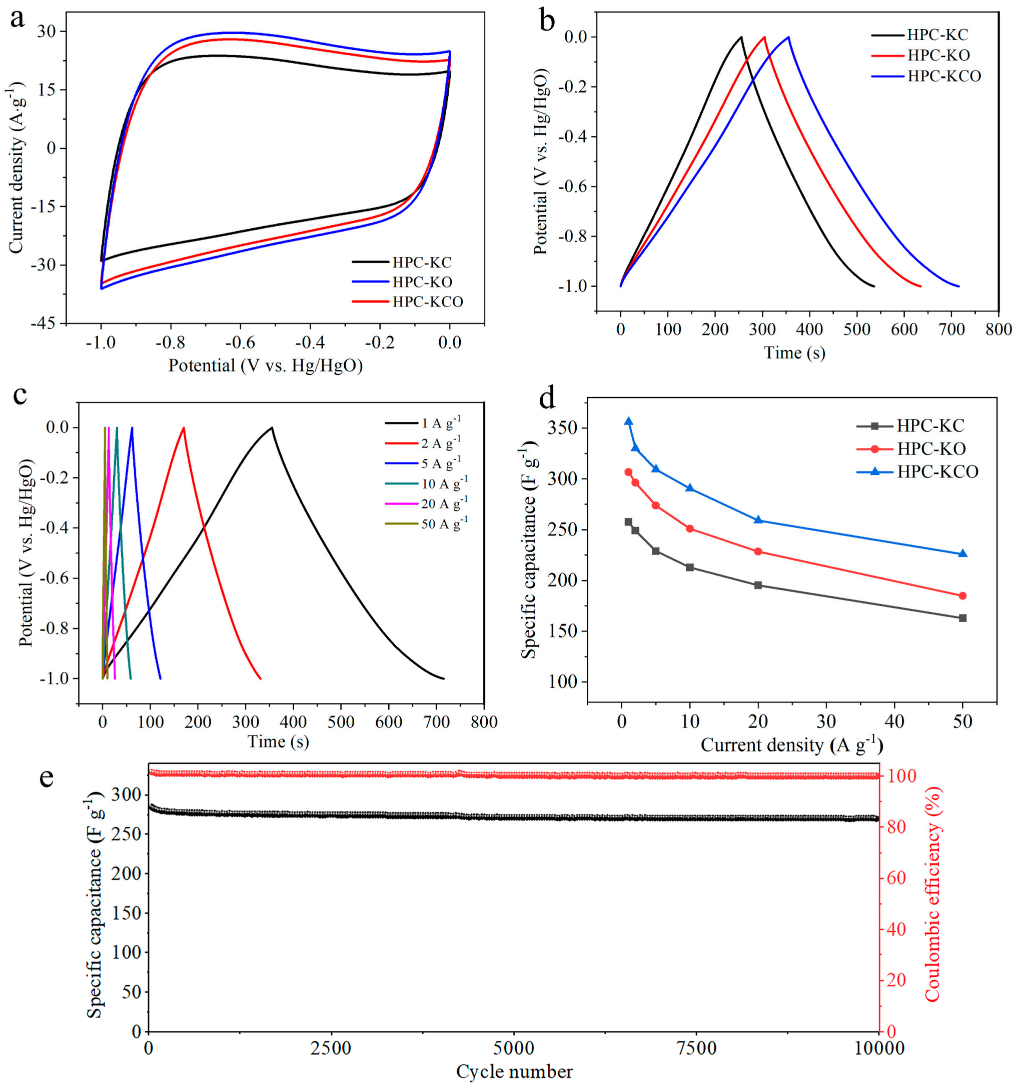

Figure S5 (a) CV curves of the HPC electrodes at 100 mV s^{-1}. (b) GCD curves at 1 A g^{-1}. (c) GCD curves of the HPC-KCO electrode at 1–50 A g^{-1}. (d) Specific capacitance of the HPC-KCO electrodes at 1–50 A g^{-1}. (e) Cycling stability of the HPC-KCO

electrode at 10 A g^{-1}. The measurement were tested in a three-electrodes system.

The electrochemical performance of the synthesized samples was evaluated in a three-electrode configuration using a 6 M KOH electrolyte. Figures S5a and S5b present the CV curves at 100 mV s^{-1} and GCD profiles at 1 A g^{-1} for the three samples, respectively. As depicted in Figure S5a, all CV curves feature a typical quasi-rectangular profile, characteristic of ideal EDLC behavior [1]. Notably, the HPC-KCO electrode exhibits the largest integrated CV area and the longest discharge time (Figure S5b), indicating that it achieves the highest specific capacitance among the investigated samples [2].

Furthermore, the GCD profiles of HPC-KCO (Figure S5c) display highly symmetrical, nearly isosceles triangular shapes across all current densities. This confirms typical EDL capacitive behavior and implies robust rate capability. Calculated from the GCD discharge curves, the specific capacitances of three samples versus current densities are plotted in Figure S5d. Although the capacitance gradually decays at higher current densities, HPC-KCO maintains a superior capacity across the board. Specifically, it delivers an impressive specific capacitance of 356F g^{-1} at 1 A g^{-1}, significantly outperforming HPC-KC (257 F g^{-1}) and HPC-KO (306 F g^{-1}). Remarkably, at 10 A g^{-1}, HPC-KCO retains 290F g^{-1} (81.6% retention), and even at an ultrahigh current density of 50 A g^{-1}, it yields 224.2 F g^{-1} (63%), manifesting exceptional rate capability.

The long-term durability of the HPC-KCO electrode was assessed via GCD cycling at 10 A g^{-1}. As illustrated in Figure S5e, the electrode demonstrates remarkable

longevity, retaining over 97% of its initial capacitance after 10,000 cycles.

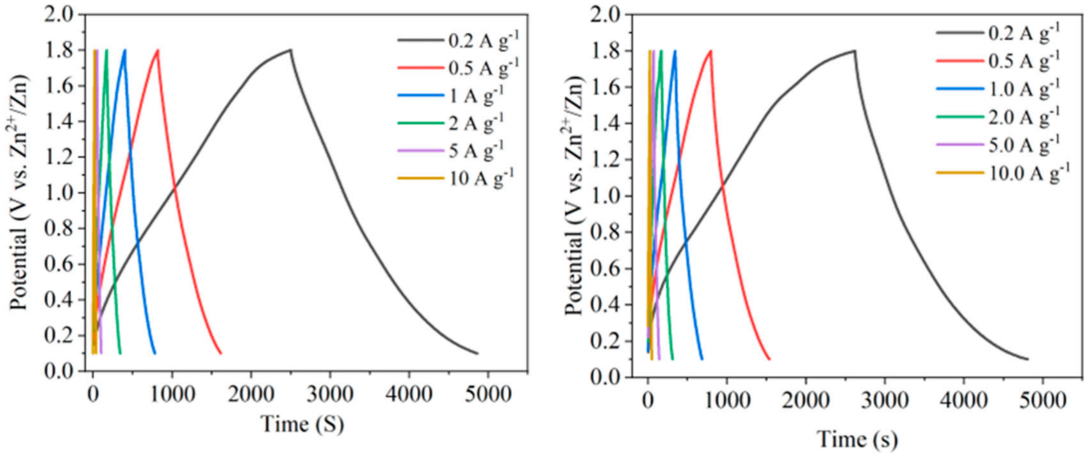

Figure S6 The GCD curves of Zn//HPC assembled with HPC-KO (a) and HPC-KC (b)

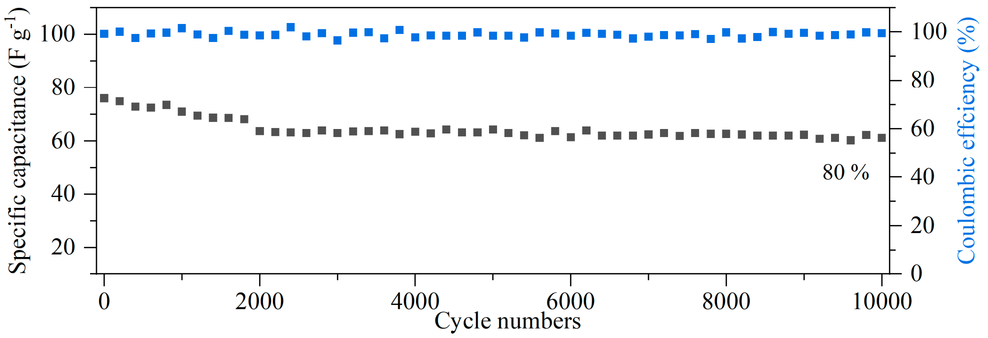

Figure S7 The cycling stability of the SSC using HPC-KCO over 10,000 cycles under -60 °C.

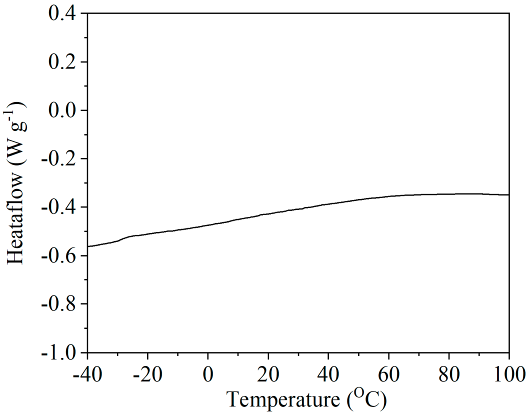

Figure S8 The DSC curves of the MPN-based electrolyte in the temperature range of -40 °C to 100 °C.

Table S1 GC/MS Library Search Report of Gaseous Products from Agarose Pyrolysis at 280℃

Retention Time	Compound name
1.64	Carbon dioxide
1.90	2-Propyn-1-ol, acetate
5.99	Furfural
7.526	Ethanone/1-(2-furanyl)-
8.772	1-Propanone
10.46	Furfuryl ethyl ether
10.62	Furyl hydroxymethyl ketone
11.10	Levoglucosenone
11.7	Hepta-2,4-dienoic acid
12.79	5-Hydroxymethylfurfural
13.71	1,4:3,6-Dianhydro-à-d-glucopyranose/α-D-Glucopyranoside
15.43	Alkanes
17.71	Alkanes

Table S2 Textural parameters of samples using BET model.

Sample	S_{BET} (m^2 g^{-1})	V_{mic} (m^3 g^{-1})	V_t (m^3 g^{-1})	D (nm)
HPC-KC	1542.2	0.64	0.78	2.01
HPC-KO	1859.6	0.57	1.08	1.17
HPC-KCO	1670.5	0.40	1.02	1.33

S_{BET}： Total BET specific surface area； V_{mi}： micropore volume； V_t： total pore volume； D： average pore size

Reference

[1] Zhuang, Z.; Wang, Z.; Xue, Y.; Su, J.; Shi, J.; Jin, H. Biomass Nanoarchitectonics of Hierarchical Porous Carbon with Ultrahigh Surface Area for Direct Air Carbon Capture and Supercapacitor. *Biomass Bioenergy* **2026**, *211*, 109160.

[2] Lu, L.; Feng, Z.; Prodhan, M. S.; Guo, X.; Zhang, X.; Zhang, W. Aged Asphalt-Derived Hierarchical Porous Carbon via Template-Activation for High Performance Supercapacitors. J. Power Sources 2026, 673, 239710.
